# Bacterial Community Sequences of Submerged Aquatic Vegetation in the Potomac River

**DOI:** 10.1128/MRA.01175-19

**Published:** 2020-01-02

**Authors:** Alexandra Alexiev, Laura E. Vann, Jenna M. Lang, Jonathan A. Eisen

**Affiliations:** a Department of Ecology and Evolutionary Biology, University of Colorado, Boulder, Colorado, USA; b Department of Evolution and Ecology, University of California, Davis, California, USA; c Department of Medical Microbiology and Immunology, University of California, Davis, California, USA; d Genome Center, University of California, Davis, California, USA; e Genomics and Bioinformatics Department, Novozymes, Davis, California, USA; Georgia Institute of Technology

## Abstract

Here, we report results from PCR and sequencing of bacterial 16S rRNA genes from leaf and root surfaces from nine submerged aquatic vegetation (SAV) samples comprising five species. Samples were from four sites along the Potomac River.

## ANNOUNCEMENT

Submerged aquatic vegetation (SAV) comprises plants that grow fully submerged in marine or freshwater most of the time and are often restricted to shallow water (1). They are globally distributed, provide habitat and food for coastal fauna, absorb wave energy and nutrients, produce oxygen, and stabilize coastal sediment (2). Human activities have heavily affected coastal regions via pollution and reduced suitable SAV habitat, particularly in the Chesapeake Bay (3). The presence of SAV is also known to influence the structure, density, and metabolic activity of sediment and rhizosphere microbial communities (4–8). Furthermore, there is evidence that different SAV species take up different amounts of nitrogen and phosphorus, which can, in turn, affect the abundance of denitrifying bacteria on their roots (8). This paper reports the results of 16S rRNA gene PCR and sequencing of samples from roots and leaves of five SAV species at four sites along the Potomac River, which feeds into the Chesapeake Bay (Table 1).

**TABLE 1:** Sample collection list

Sample description	No. of reads per sample	Raw count in bp	Trimmed count in bp (% of raw count)	Merged count in bp (% of raw count)
Kit.control	25	140	87 (62.14)	54 (38.57)
Kit.control	22	67	48 (71.64)	31 (46.27)
Site.P1.UnkSps.Leaf	11	64	46 (71.88)	28 (43.75)
Site.P1.UnkSps.Leaf	2,815	3,466	3,293 (95.01)	2,991 (86.3)
Site.P1.UnkSps.Leaf	9,023	12,438	11,785 (94.75)	10,174 (81.8)
Site.P1.UnkSps.Root	84,120	102,951	97,670 (94.87)	91,468 (88.85)
Site.P1.UnkSps.Root	100,126	130,084	120,239 (92.43)	110,333 (84.82)
Site.P1.UnkSps.Root	90,173	112,716	106,847 (94.79)	99,397 (88.18)
Site.P1.Vallisneria_sps_unk.Leaf	104	437	366 (83.75)	261 (59.73)
Site.P1.Vallisneria_sps_unk.Leaf	6,982	13,335	12,735 (95.5)	12,145 (91.08)
Site.P1.Vallisneria_sps_unk.Leaf	16,302	24,985	23,611 (94.5)	22,038 (88.2)
Site.P1.Vallisneria_sps_unk.Root	22,369	32,515	31,401 (96.57)	30,335 (93.3)
Site.P1.Vallisneria_sps_unk.Root	14,430	25,714	24,428 (95)	22,771 (88.55)
Site.P1.Vallisneria_sps_unk.Root	46,096	67,889	64,803 (95.45)	61,161 (90.09)
Site.P1.Ruppia_maritima.Leaf	1,237	4,174	4,045 (96.91)	3,813 (91.35)
Site.P1.Ruppia_maritima.Leaf	34	263	230 (87.45)	147 (55.89)
Site.P1.Ruppia_maritima.Leaf	280	882	811 (91.95)	714 (80.95)
Site.P1.Ceratophyllum_demersum.Leaf	4,041	10,431	10,180 (97.59)	9,805 (94)
Site.P1.Ceratophyllum_demersum.Leaf	2,037	4,099	3,908 (95.34)	3,627 (88.48)
Site.P1.Ceratophyllum_demersum.Leaf	6,237	21,693	21,296 (98.17)	20,790 (95.84)
Site.P2.Potamogeton_perfoliatus.Leaf	20,481	50,697	49,585 (97.81)	48,095 (94.87)
Site.P2.Potamogeton_perfoliatus.Leaf	13,513	37,567	36,566 (97.34)	35,302 (93.97)
Site.P2.Potamogeton_perfoliatus.Leaf	16,021	38,842	37,440 (96.39)	35,605 (91.67)
Site.P2.Potamogeton_perfoliatus.Root	39,788	46,487	45,037 (96.88)	42,876 (92.23)
Site.P2.Potamogeton_perfoliatus.Root	11,998	14,095	13,611 (96.57)	12,944 (91.83)
Site.P2.Potamogeton_perfoliatus.Root	31,799	35,214	34,276 (97.34)	32,998 (93.71)
Site.P2.Vallisneria_sps_unk.Leaf1	16,774	60,180	59,031 (98.09)	57,467 (95.49)
Site.P2.Vallisneria_sps_unk.Leaf1	14,042	44,402	43,358 (97.65)	41,775 (94.08)
Site.P2.Vallisneria_sps_unk.Leaf1	20,938	84,103	81,431 (96.82)	77,746 (92.44)
Site.P2.Vallisneria_sps_unk.Root	98,013	128,459	120,010 (93.42)	112,773 (87.79)
Site.P2.Vallisneria_sps_unk.Root	22,709	33,065	30,031 (90.82)	27,348 (82.71)
Site.P2.Vallisneria_sps_unk.Root	26,191	40,148	37,109 (92.43)	34,564 (86.09)
Site.P2.Vallisneria_sps_unk.Leaf2	11,863	37,872	36,591 (96.62)	34,886 (92.12)
Site.P2.Vallisneria_sps_unk.Leaf2	172	720	663 (92.08)	550 (76.39)
Site.P2.Vallisneria_sps_unk.Leaf2	5,914	20,316	19,772 (97.32)	19,030 (93.67)
Site.P2.Myriophyllum_spicatum.Leaf	33,577	119,521	117,639 (98.43)	115,176 (96.36)
Site.P2.Myriophyllum_spicatum.Leaf	32,088	106,020	103,336 (97.47)	98,959 (93.34)
Site.P2.Myriophyllum_spicatum.Leaf	38,571	113,778	110,832 (97.41)	106,517 (93.62)
Site.P3.Myriophyllum_spicatum.Leaf	8,767	26,684	26,169 (98.07)	25,325 (94.91)
Site.P3.Myriophyllum_spicatum.Leaf	4,555	10,840	10,498 (96.85)	10,095 (93.13)
Site.P3.Myriophyllum_spicatum.Leaf	452	1,534	1,466 (95.57)	1,336 (87.09)
Site.P3.Myriophyllum_spicatum.Root	18,799	23,568	22,105 (93.79)	20,838 (88.42)
Site.P3.Myriophyllum_spicatum.Root	42,702	51,778	47,966 (92.64)	45,262 (87.42)
Site.P3.Myriophyllum_spicatum.Root	16,185	18,642	17,771 (95.33)	17,138 (91.93)
Site.P3.Vallisneria_sps_unk.Leaf	1,692	3,746	3,657 (97.62)	3,563 (95.11)
Site.P3.Vallisneria_sps_unk.Leaf	53	259	165 (63.71)	107 (41.31)
Site.P3.Vallisneria_sps_unk.Leaf	12,516	20,122	19,408 (96.45)	18,394 (91.41)
Site.P3.Vallisneria_sps_unk.Root	41,072	49,833	47,140 (94.6)	44,681 (89.66)
Site.P3.Vallisneria_sps_unk.Root	34,861	41,769	40,052 (95.89)	37,960 (90.88)
Site.P3.Vallisneria_sps_unk.Root	7,965	9,528	8,991 (94.36)	8,594 (90.2)
Site.P4.Myriophyllum_spicatum.Leaf	7,395	10,019	9,588 (95.7)	9,240 (92.22)
Site.P4.Myriophyllum_spicatum.Leaf	6	29	25 (86.21)	12 (41.38)
Site.P4.Myriophyllum_spicatum.Leaf	4,404	6,691	5,855 (87.51)	5,498 (82.17)
Site.P4.Myriophyllum_spicatum.Root	2,935	4,721	4,510 (95.53)	4,254 (90.11)
Site.P4.Myriophyllum_spicatum.Root	4,988	9,583	9,088 (94.83)	8,510 (88.8)
Site.P4.Myriophyllum_spicatum.Root	122	411	314 (76.4)	221 (53.77)

Triplicate leaf and root samples were taken from an individual of each visually distinct species of SAV identified at each site and stored in a sterile tube of Xpedition lysis/stabilization solution (Zymo Research, Irvine, CA) at room temperature until DNA extraction. Sites were named P1 to P4, with P1 being the most freshwater site (0.18 ppt) and P4 being the most marine (8.08 ppt) and closest to the Chesapeake Bay. Briefly, we processed the samples as follows. DNA was extracted using the PowerSoil DNA isolation kit (MoBio Laboratories, Carlsbad, CA). PCR amplification of the V4 region of the 16S rRNA gene was conducted with the bacterial/archaeal primers 515F/806R (9) and custom barcodes (Invitrogen, Carlsbad, CA) (10). Samples were sequenced on a MiSeq instrument (Illumina, San Diego, CA) at the University of California (UC) Davis Genome Center Sequencing Core, using the MiSeq 500-cycle v2 kit for 250-bp paired-end sequencing. This yielded 1,070,385 reads for 56 samples, ranging from 6 reads per sample to 100,126 reads per sample.

Sequence processing was done on a lab server (Linux 3.2.0-29-generic number 46-Ubuntu, 16 central processing units [CPUs] and 48 GB of RAM). Demultiplexing used a custom script which automates quality assessment and trimming (https://github.com/gjospin/scripts/blob/master/Demul_trim_prep.pl). The script trims bases from the right side of the reads that are below Q20 and then discards reads less than 35 bp long. Once sequences are trimmed, they are merged. Open-reference operational taxonomic units (OTUs) were picked at a 97% cutoff with QIIME version 1.9.0 (11) and the Greengenes database (12). Reads identified as being from chloroplasts or mitochondria, flagged as chimeric, or not assigned a taxonomy were removed from further processing. Detailed protocols and workflow documentation are available online (https://doi.org/10.6084/m9.figshare.5860926.v3).

There were 1,070,385 reads total for 56 samples after the above sequence-processing steps. The average number of reads per sample was 19,114 and ranged from 6 to 100,126. Sample-specific metrics are listed in Table 1. We identified 6,634 microbial OTUs total from our samples (*n* = 40). OTUs assigned to the genera *Methylotenera* (2.2% of all sequences), *Sulfurimonas* (1.4%), and *Sulfuricurvum* (2.9%) and the family *Rhodocyclaceae* (1.4%) are the most common among all the samples. Leaf samples were dominated by Methylotenera mobilis (3.5% of leaf-associated sequences) and the genus *Planctomyces* (2.2%), whereas root samples were dominated by Sulfuricurvum kujiense (6.8% of root-associated sequences), the genus *Sulfurimonas* (6.0%), and the family *Rhodocyclaceae* (5.7%).

### Data availability.

Sequences from this data set are available through NCBI under the accession number PRJNA305164. Detailed protocols and workflow documentation are available on FigShare (https://doi.org/10.6084/m9.figshare.5860926.v3).
